# *KRAS* mutation status concordance between the primary tumor and the corresponding metastasis in patients with rectal cancer

**DOI:** 10.1371/journal.pone.0239806

**Published:** 2020-10-01

**Authors:** Peter Jo, Markus Bernhardt, Manuel Nietert, Alexander König, Azadeh Azizian, Markus A. Schirmer, Marian Grade, Julia Kitz, Kirsten Reuter-Jessen, Michael Ghadimi, Philipp Ströbel, Hans-Ulrich Schildhaus, Jochen Gaedcke

**Affiliations:** 1 Department of General, Visceral and Pediatric Surgery, University Medical Center Goettingen, Goettingen, Germany; 2 Department of Medical Statistics, University Medical Center Goettingen, Goettingen, Germany; 3 Department of Gastroenterology and Gastrointestinal Oncology, University Medical Center Goettingen, Goettingen, Germany; 4 Department of Radiotherapy and Radiation Oncology, University Medical Center Goettingen, Goettingen, Germany; 5 Department of Pathology, University Medical Center Goettingen, Goettingen, Germany; 6 Department of Pathology, University Medical Center Duisburg-Essen, Essen, Germany; Virginia Commonwealth University, UNITED STATES

## Abstract

**Introduction:**

Oncogenic mutation within the *KRAS* gene represents a negative predictor for treatment response to anti-epidermal growth factor receptor (*EGFR*) in patients with colorectal cancer. Recently, we have shown no relevant heterogeneity for *KRAS* mutation status within and between pre- and posttherapeutic samples from the primary tumor in patients with locally advanced rectal cancer. The aim of this study was to evaluate the intertumoral heterogeneity of *KRAS* mutation status between the primary tumor and the corresponding metastasis or local recurrence in the similar cohort and to evaluate the ideal representative tissue for *KRAS* mutation testing.

**Materials and methods:**

*KRAS* mutation status was analyzed from 47 patients with locally advanced rectal cancer, which were enrolled in the CAO/ARO/AIO-94 or CAO/ARO/AIO-04 trial. Mutations in *KRAS* codons 12, 13, and 61 were analyzed by using the *KRAS* RGQ PCR Kit (therascreen® *KRAS* test). Six patients needed to be excluded due to incomplete follow up data. 11 patients showed a relapse of the disease during the follow up presented by distant metastases or local recurrence. DNA from representative areas of metastatic tissue was obtained from formalin-fixed paraffin-embedded specimens.

**Results:**

The mean patient age was 64.13 ± 10.64 years. In total, 19 patients showed a *KRAS* mutation (46.34%) in the primary tumor. Of the eleven patients with a metastatic disease or local recurrence, five patients showed a *KRAS* mutation whereas six patients had a *KRAS* wildtype status. Metastatic localizations included the liver (n = 2), lung (n = 4), local recurrence (n = 1), liver + lung (n = 3), lung + local recurrence (n = 1). For these eleven patients with paired data available for the primary tumor and metastatic tissue, a significant *KRAS* mutation status concordance was detected in 81.18% (9/11) of the patients (*p* = 0.03271). Only two patients showed intertumoral heterogeneity, which harbored in one patient a *KRAS* G12C mutation status in the primary tumor, but a G12V *KRAS* mutation status in the corresponding lung lesion, and in the other patient a G12A mutation in the primary lesion and a WT in the lung metastasis.

**Conclusions:**

We show a significant concordance of the *KRAS* mutation status between tumor samples obtained from the primary tumor and the corresponding metastasis and/ or local recurrence in patients with rectal cancer indicating no relevant intertumoral heterogeneity. Our data suggest that sampling either the primary (pre- or posttherapeutical tumor tissue) or metastatic lesion may be valid for the initial evaluation of KRAS mutation status predicting the response to anti-EGFR treatment and guiding clinical decisions.

## Introduction

In patients with metastatic colorectal cancer (CRC) the clinical implementation of targeted therapy strategies against the epidermal growth factor receptor (EGFR) has become a well-established oncological therapy option. FOLFIRI in combination with the monoclonal antibody cetuximab against the EGFR has been shown to reduce the risk of progression in patients with *KRAS* wild-type (WT) status [1] and mutated *KRAS* status is associated with resistance to cetuximab and a shorter survival [2]. More clinical evidence came up from different authors and the data revealed the therapeutical benefit of monoclonal antibodies against the EGFR in *KRAS* wild-type patients. Hence, patients with activating mutations in the *KRAS* gene do not respond significantly to an anti-EGFR therapy [2–4]. Thus, *KRAS* mutation turned out to be a negative response predictor to monoclonal antibody therapy against *EGFR*, allowing an individual stratification based on the *KRAS* mutation status. Patients with a mutated KRAS status can be spared from anti-EGFR treatment to avoid unnecessary adverse effects and costs [5, 6]. Therefore, the reliable and valid detection of the KRAS mutation status is mandatory before initialization anti-EGFR therapy.

Until now it is still unclear whether the KRAS mutation status should be determined by analyzing the primary tumor or metastatic tissue. Additionally intratumoral and intertumoral heterogeneity [7–9] might be a challenging phenomenon in the diagnosis of a potential activating KRAS mutation status at least for individual patients with therapeutic consequences [10, 11].

Currently preoperative chemoradiotherapy (CRT) is the standard treatment modality in patients with locally advanced rectal cancer in the lower and middle part of the rectum. Patients with colon cancer do not receive a neoadjuvant CRT by default. However most of the published works evaluating intra- and intertumoral heterogeneity do not distinguish between colon- and rectal cancer although there are known differences in oncogenic molecular pathways and therapy options.

In a previously published work we have shown the valid assessment of KRAS mutation status for therapeutic decisions in pre-therapeutic (treatment naïve) biopsies as well as in post-therapeutic (after preoperative CRT) residual tumor tissue in patients with locally advanced rectal cancer [12]. We assessed a homogenous KRAS mutation status between several pre- and post-therapeutic tissue samples of the primary tumor indicating no relevant intratumoral heterogeneity and concluded, that discordance is a very rare event [12].

In this study, we focused on the assessment of a potential *KRAS* mutation heterogeneity between the primary tumor and the corresponding recurrence site in the same patient cohort (referred to as intertumoral heterogeneity). Previous controversial reports have evaluated intertumoral heterogeneity, showing both discordance and concordance in the *KRAS* mutation status between the primary tumor and the matched metastases [11, 13], thus intertumoral heterogeneity might implicate a clinical problem in CRC. *KRAS* mutation status heterogeneity may differ significantly between studies, ranging from 0% to 30% [14, 15], potentially caused by the utilized mutation testing methodology or sampling error [10, 16–18].

Until today, there is still no definitive testing solution for *KRAS* mutations between the primary tumor or the metastatic sites in patients with metastatic CRC [19]. Thus, the aim of this study was to evaluate the intertumoral heterogeneity of the *KRAS* mutation status between the primary lesion and the corresponding metastatic tissue, and hereby, to evaluate the ideal representative tissue for *KRAS* mutation testing in a cohort of patients with locally advanced rectal cancer treated with preoperative CRT within a prospective, randomized clinical trial setting.

## Materials and methods

### Patients and treatment

Analyses of the *KRAS* mutation status were performed from 47 patients with locally advanced rectal cancer as previously described to evaluate the intratumoral heterogeneity in pre-, and post-therapeutic tumor samples [12]. In this study, out of the 47 patients, six patients were excluded due to missing follow up data. 11 patients showed recurrence including liver-, lung-, and/ or local recurrence during the follow up. DNA from one area of metastatic tissue was obtained from formalin-fixed paraffin-embedded specimens from 11 metastatic rectal cancer patients. Mutations in *KRAS* codons 12, 13, and 61 were analyzed by using the *KRAS* RGQ PCR Kit (therascreen® *KRAS* test, QIAGEN, Hilden, Germany) according to the manufacture`s instructions.

Patients included in this analysis were treated at the Departments of General, Visceral and Pediatric Surgery and Radiotherapy and Radiation Oncology, University Medical Center Goettingen, and were enrolled or treated according to the trial guidelines of the CAO/ARO/AIO-94 [20] or CAO/ARO/AIO-04 [21] (EudraCT-Number 2006-002385-20—NCT00349076) of the German Rectal Cancer Study Group.

Patients were treated preoperatively with CRT followed by surgical resection and standardized pathologic workup. Preoperative CRT included an irradiation of the presacral space with an overall dose of 50.4 Gy (single dose of 1.8 Gy) accompanied by either 5-fluorouracil or a combination of an intravenous infusion of oxaliplatin and a continuous infusion of 5-fluorouracil. Within four to six weeks after completion of preoperative CRT, surgery was carried out, including total mesorectal excision. All patients were followed-up according to the trial protocols and gave written informed consent either from the patients or their legal representatives. Both studies were conducted in accordance with the ethical principles of the Declaration of Helsinki (Seoul, 2008) and were approved by the University of Goettingen Ethics Committee in Goettingen, Germany (application number 20/9/95, 9/8/08).

### Ascertainment of pre- and post-therapeutic and metastatic tumor biopsies

Tumor biopsies were collected prior to preoperative CRT during diagnostic procedures. After surgery, residual tumor was taken for analyses from the resected specimens [12, 22]. Patients presenting metastases or local recurrence during follow-up were subjected to needle biopsy or surgical biopsy and resulting tumor tissue was referred to histopathological confirmation and *KRAS* mutation analysis. The mutation status was assessed in formalin-fixed-paraffin-embedded (FFPE) tissue samples (4% buffered formalin) from pre-therapeutic tumor biopsies, post-operative resected specimens and metastatic or local recurrence tumor tissue.

### Tumor DNA preparation and isolation

FFPE slides from pretherapeutic-, resected-, and metastatic specimens were independently and blinded reevaluated by two experienced gastrointestinal pathologists (J.K., P.S.). Tumorregression grading (TRG) was assessed in percentage of regression in accordance with the Dworak Grading system [23]. In discordant cases, slides were reassessed by both pathologists and a final decision was made. We summerized the respective tumor regression grades (TRG) and relevant clinical data in Table 1. Microdissection for tumor cell enrichment to achieve a content of 70–80% was performed manually as recently reported by Hunt and colleagues [24]. Briefly, FFPE tissue slices were deparaffinized and stained with Haematoxylin. Representative tumor areas were identified using a microscope at a 40x magnification. Dissection was performed using a pointed surgical blade. Tumor tissue was transferred to a tube and DNA extraction was performed subsequently by using the QIAGEN AllPrep DNA/ RNA FFPE Kit (QIAGEN, Hilden, Germany) according to the manufacturer`s instructions.

**Table 1:** Clinical data with tumor regression grading.

ID	Gender	uT	uN	cM	Tumor height (cm)	pT	pN	TRG (%)	TRG (Dworak)
1	m	3	1	0	5	3	1	60	3
2	m	3	1	0	3	3	1	35	2
3	f	3	1	0	12	3	1	20	1
4	m	3	0	0	4	3	0	15	1
8	m	3	0	0	5	3	0	30	2
9	f	3	0	0	7	3	0	40	2
11	m	3	0	0	12	3	0	40	2
12	m	3	1	0	4	3	1	90	3
13	m	3	1	0	4	3	1	70	3
14	f	3	1	0	1	3	1	55	3
15	m	2	1	0	3	3	0	20	1
16	m	3	0	0	8	3	1	10	1
17	f	3	1	0	9	3	1	70	3
18	f	3	0	0	8	3	0	40	2
19	m	3	1	0	8	3	1	70	3
20	m	3	1	0	6	3	1	40	2
21	m	3	0	0	11	3	0	20	1
22	m	4	1	0	8	3	1	45	2
23	m	2	1	0	5	2	0	40	2
24	m	3	1	0	11	3	1	80	3
25	m	3	1	0	7	3	1	45	2
26	f	3	1	0	11	3	0	40	2
27	m	3	0	0	10	3	0	70	3
28	m	3	1	0	4	3	1	70	3
30	m	3	0	0	7	3	0	80	3
31	m	3	1	0	8	3	0	45	2
32	m	3	1	0	8	3	1	50	3
33	f	3	1	0	4	3	1	70	3
34	f	3	1	0	8	3	1	45	2
36	f	3	1	0	5	3	1	70	3
37	m	3	1	1	8	3	1	70	3
38	m	3	1	1	9	3	1	45	2
39	m	3	1	0	6	3	1	70	3
40	f	3	1	1	5	3	1	90	3
41	f	3	1	0	5	3	1	35	2
42	f	3	1	0	3,5	3	1	70	3
43	f	3	1	0	9	3	1	95	3
44	f	3	1	0	11	3	1	30	2
45	m	3	1	0	10	3	0	10	1
46	m	3	0	0	1	3	0	70	3
47	m	3	0	0	3	3	0	50	3

Relevant clinical data and TRG in % and TRG according to Dworak for the analyzed patients (m = male, f = female, tumor height in cm with respect to the anocutaneous line, ultrasonographic/clinical (u/c) and pathological (p) TNM stage: T = tumor; N = lymph node, M = metastasis, TRG = tumor regression grading).

### Mutation analysis

#### Therascreen® *KRAS* test

The *KRAS* Pyro Kit 24, V1 (QIAGEN, Hilden, Germany) (cover mutations in *KRAS* codons 12, 13, and 61 of the human *KRAS* gene) and the *RAS* Extension Pyro Kit 24, V1 (QIAGEN, Hilden, Germany) (cover mutations in *KRAS* codons 59, 61, 117 and 146 of the human *KRAS* gene) have been conducted for the validation detection of mutations of the *KRAS* gene in genomic DNA of rectal cancer specimen. The PyroMark Q24 MDx platform has been used to run the Therascreen1-assay with the Software Q24, Version 2.0.7 with following PlugIns, *KRAS* PlugIn v1.2.0 and *RAS* Extention PlugIn v.1.2.1.2. We amplified regions of interest in the extracted DNA using primers in the *KRAS* Pyro assay (QIAGEN, Hilden, Germany). We subsequently immobilized, washed, and denatured the amplified products using the vacuum workstation and subjected those products to pyrosequencing using the PyroMark Q24 Pyrosequencer (QIAGEN, Hilden, Germany) to detect and quantify the *KRAS* mutations. Initial DNA input for each specimen was 100 ng.

### Pre-processing and statistical analysis

The workflow for the pre-processing and the analysis of the data was implemented using KNIME 3.6.2 [25]. KNIME nodes were mostly used for the pre-processing of the data, while we used the R-plugin nodes in KNIME to perform the final statistical tests with R version 3.6.1 [26]. The global significance level was set to α = 5%. In case of count data, we used the Fishers Exact test or Chi-Square test depending on the available number of samples for the comparison. The impact of the 'mutation status' and 'clinical parameters' on survival was determined using Kaplan-Meier analysis and assessed for statistical significance using the log rank test and where applicable for the continuous data values using a Cox proportional hazard model [27]. The survival analysis was performed using the R package ‘survival’.

## Results

### Clinicopathological characteristics and results from mutation analysis

41 patients with metastatic rectal cancer were included: 28 (68.3%) male and 13 (31.7%) female patients. The mean age was 64.13 ± 10.64 years. Clinical findings together with data with tumor regression grading is listed in Table 1. *KRAS* mutation was found in 19 patients (46.34%)– 31.70% of mutated cases were in codon 12, compared with 7.32% in codon 13, 4.88% in codon 146 and 2.44% in codon 61. In codon 12, 38.46% contained the *KRAS* c.35G>A (G12D) mutation, followed by 30.77% with c.35G>T (G12V), 15.38% with c.34G>T (G12C), and 15.38% with c.35G>C (G12A). In codon 13, 100% of the mutations consisted of c.38G>A (G13D) and in codon 146, 100% were c.436G>A (A146T). The only case with a mutation in codon 61 was c.182A>T (Q61L).

### Concordance of *KRAS* mutation status between primary and matched metastatic lesion

The concordance between primary and metastatic tissues in the same patients was analyzed in 11 matched samples. We examined available metastatic tumor samples for each patient, including lung-, liver-, and bone metastases or local recurrence. The *KRAS*- and WT status of the metastatic sites and their corresponding primary tumors are presented in Table 2. We observed in 81.18% a significant concordance of *KRAS* mutation status between the primary tissue and the corresponding metastatic samples (*p* = 0.03271).

**Table 2:** *KRAS* mutation status in patients with metastasized rectal cancer.

ID	Primary Tumor (Preoperative)	Primary Tumor (Postoperative)	Liver	Lung	Local Recurrence
3	G12D	G12D	-	G12D	-
4	WT	WT	WT	WT	-
12	WT	WT	-	WT	WT
13	WT	WT	WT	-	-
17	G12C	G12C	-	G12V	-
21	WT	WT	WT	-	-
26	G12A	G12A	-	WT	-
32	WT	WT	WT	WT	-
40	G12V	G12V	-	-	G12V
46	G12D	G12D	-	G12D	-
47	G12V	G12V	G12V	G12V	-

*KRAS* mutation and Wildtype status distribution of the patients with metastasized rectal cancer.

Only two patients showed intertumoral heterogeneity. The first discordant patient (Patient 17), a 79-year-old male patient, harboring a *KRAS* G12C mutation in the primary tumor showed a G12V *KRAS* mutation in the corresponding lung lesion. To confirm these results, we reevaluated both tumor tissues using an alternative therascreen® *KRAS* test revealing the same result as the initial test. The histopathologic workup in this case showed initially a ypT3b, ypN1, cM0 status of the primary tumor. The patient did not receive an adjuvant chemotherapy. One year and seven months after the primary resection of the rectal cancer the patient developed lung metastasis, histopathologically confirmed by a bronchoscopy biopsy.

The second discordant patient (Patient 26) showed a G12A mutation in the primary lesion and a wild-type situation in the synchronous liver metastasis as well as in the metachronous lung metastasis. These findings were again confirmed by the additional therascreen® *KRAS* test. After an intensified preoperative CRT (neoadjuvant CRT followed by two additional cycles of neoadjuvant CT) the patient showed at the timepoint of surgery liver metastases in the right lobe and together with the rectum resection the patient was referred to an atypical liver resection for histopathological confirmation of metastasis (ypT3b ypN0 (0/35) ypM1 (HEP) G3, R0). After 6 cycles of chemotherapy using the FOLFOX6 protocol an extended hemihepatectomy of the right lobe was conducted without confirmation of any vital tumor-cells in the histopathological workup. Ten months after the rectum resection, the patient developed lung metastasis and received 6 cycles of chemotherapy using the FOLFIRI protocol. Subsequently, the patient underwent a two-stage atypical lung resection of the upper lobe right and the lower lobe left and histopathological examination confirmed metastasis of the rectal cancer. The patient received chemotherapy using different protocols.

### *KRAS* mutation status and survival

**Fig 1:**
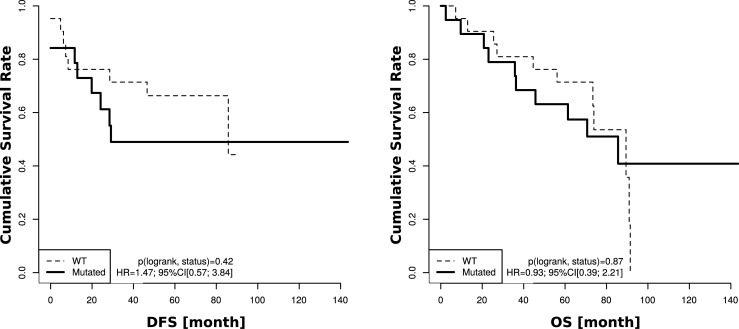
Caplan-meier curves for DFS and OS. DFS and OS Data was available for 40 patients (*KRAS* mutated n = 19, *KRAS* wild-type n = 21). No significant correlation between *KRAS* mutated and wild-type patients was found for DFS and OS.

For 40 patients *KRAS* mutation and wild-type status was correlated to survival parameters. However, we did not find any significant correlation between *KRAS* mutated and wild-type patients with disease free survival (DFS) (*p* = 0.42) or overall survival (OS) (*p* = 0.87) (Fig 1).

## Discussion

In this study, we were able to prove that *KRAS* mutation status is statistically significant homogeneous between the primary tumor and the corresponding metastatic tissue in patients with locally advanced rectal cancer by applying a sensitive pyrosequencing method. Our results implicate that *KRAS* mutation status remains consistent in the oncological evolution to metastatic disease. Regarding rectal cancer solely, this study is to our knowledge one of the largest cohort for the specific evaluation of intra- [12] and intertumoral *KRAS* mutation status heterogeneity in patients with locally advanced rectal cancer who were treated preoperatively with CRT within a clinical study setting. Most of the published data dealing with intertumoral heterogeneity in CRC analyzed colon- and rectal together as one entity, although many differences exist in aetiology, genetics, anatomy, treatment strategies and treatment response between colon and rectal cancer. This work focuses on an exclusive sample set of patients with locally advanced rectal cancer who were enrolled or treated according to the trial guidelines of the CAO/ARO/AIO-94 or CAO/ARO/AIO-04 of the German Rectal Cancer Study Group.

The investigated cohort in this study harbored a *KRAS* mutation frequency of 46% which is in line with current data for colon and rectal cancer. Moreover the distribution of the single hotspot mutations observed were similar to what has been published in the past [3, 5, 6, 28, 29]. Thus, our findings make us confident that our results display a reliable data set.

Our data is in concordance with a meta-analysis from Cheng-Bo Han and colleagues who analyzed the *KRAS* mutation status between primary and metastatic tumor samples in patients with colorectal cancers. Analyzing 19 publications *KRAS* mutation status turned out to be highly concordant in primary and distant metastatic tumors [15]. Important to mention, Han and colleagues did not distinguish between colon and rectal cancer in their analyses. Additionally, it remains unclear if the patients with rectal cancer received a preoperative CRT.

In a former study our results demonstrated the reliable detection of *KRAS* mutation status for therapeutic decisions in pre-therapeutic biopsies as well as in post-therapeutic residual tumor tissue in patients with locally advanced rectal cancer who were treated preoperatively with chemoradiotherapy. We have reported that intratumoral *KRAS* heterogeneity is a very rare event. Additionally, we pointed out the importance to assess the *KRAS* mutation status with a sensitive technical valid method [12]. Therefore, our results demonstrate the reproducibility of *KRAS* testing in the primary biopsy as well as in the resected specimen within the same primary tumor as well as in the corresponding metastatic tissue. However, some minor discrepancy occurred. Two patients showed intertumoral heterogeneity, which harbored in one patient a *KRAS* G12C mutation status in the primary tumor, but a G12V *KRAS* mutation status in the corresponding lung lesion, and in the other patient a G12A mutation in the primary lesion and a WT in the lung metastasis. The minor discrepancy occurred only in the corresponding lung metastases, possibly indicating a distinct pattern and molecular dynamics of lung metastases and therefore should be taken into account. Nevertheless, this finding has to be proven in a larger cohort. Another assumption for *KRAS* mutation status heterogeneity could be DNA degradation [30] as well the possibility that discordance is based on the existence of a secondary tumor next to the colorectal cancer entity [31].

Previous controversial reports have evaluated intertumoral heterogeneity, showing both discordance and concordance in the *KRAS* mutation status between the primary tumor and the matched metastases in patients with colorectal cancer [11, 13, 14]. Docs et al. analyzed the *KRAS* status in 18 out of 665 patients from at least two sequential samples obtained at different time points and observed intertumoral heterogeneity in six cases [32].

Currently *KRAS* mutation status concordance between the primary and metastatic tumor site has become more and more obvious. Knijn et al. observed a high concordance of *KRAS* mutation status of 96.4% between primary colorectal tumors and their corresponding liver metastases. Analyzing 305 patients, eleven patients showed a discordant *KRAS* mutation status. The authors suggested that tissue from the primary tumor or from the liver metastasis can be applied for *KRAS* mutation testing [14]. However, their analyses concentrated only on liver metastases and the *KRAS* mutation status has only been investigated for codons 12 and 13. Vakiani et al. examined the *KRAS* mutation status heterogeneity of a cohort of 84 patients with matched primary and metastatic tumors. After reevaluating FFPE tissue from initially heterogenous cases, they could detect a concordance rate of 98.8% [31]. In a work by Miglio et al. forty-five primary tumors and matched metastases have been analyzed by applying the same highly sensitive TheraScreen**®** technique used in our study and the authors detected in twenty-eight patients with metastatic disease no single case of intertumoral heterogeneity [33]. Paliogiannis and colleagues reported a *KRAS* concordance rate of 90.3% between primary tumors and metastatic sites in patients with colorectal cancer and confirmed as well a high *KRAS* homogeneity [34]. After next-generation sequencing technology has been established and more widely available, more studies were conducted to evaluate *KRAS* heterogeneity between primary and metastatic tumors. Brannon et al. performed deep coverage, targeted next-generation sequencing in 69 matched primary and metastatic tumors and detected a concordance rate of 100% for *KRAS* mutation status in patients with colorectal cancer [35]. Crumley et al. performed next-generation sequencing in sixteen patients with rectal cancer, evaluating driver mutations in comparison to colon cancer. Here they could demonstrate a concordance rate of 93% for the KRAS mutation status between the primary tumor and metastasis. The authors focused similarly to this study on rectal cancer solely. However, not all patients received preoperative chemoradiotherapy [36].

In our study, although two of 11 patients showed a heterogeneous *KRAS* mutation status no case with discordance in the *KRAS* status between the primary and metastatic tumors had a clinical significance and thus represented no alteration in clinical decision-making. In both discrepant cases the primary tumor harbored a *KRAS* mutation, thus the patients were initially stratified to receive no *EGFR*-antibody therapy because of the presence of a *KRAS* mutation. In general, patients with heterogenous *KRAS* mutation status between the primary- and metastatic tumor would not display a clinical dilemma like the first heterogenous case (Patient 17) harboring a *KRAS* G12C mutation status in the primary tumor, but a G12V *KRAS* mutation status in the corresponding lung lesion.

The second patient (patient 26) who showed intertumoral heterogeneity between the primary tumor (G12A) and the corresponding lung metastasis (WT) has been initially in a synchronous metastasized situation and showed primarily liver metastases. During the follow-up, the patient developed lung metastases. There are some data about patients with synchronous metastasized colorectal cancer which might have a higher rate of discordance in the *KRAS* mutation status [37–39]. The above mentioned second patient 26 received adjuvant chemotherapy before resection of the lung metastasis. Although the rate of mutations has been shown to accumulate after chemotherapy [40], data specifically changes from *KRAS* mutation in the primary tumor to WT in the metastasis seems to be a rare event. A possible explanation could be the presence and coexistence of several independent subclones originated in a limited number of patients [34, 41].

We confirmed a high concordance in the *KRAS* mutation status between the primary tumor and the metastatic lesion, which seems to maintain stable during carcinogenesis. These findings go along with Vogelstein´s proposed colorectal carcinogenic model [42] in which *KRAS* mutations evolve early and maintain consistent during carcinogenesis [41]. In our study the frequency of *KRAS* mutations in metastatic or recurrent disease is much similar to that of patients without local or systemic relapse. This suggests that *KRAS* mutations alone are not sufficient for changes in the epithelial morphology of colorectal cancer cells regarded as necessary for metastasis evolvement [43]. Thus, *KRAS* mutations alone do not seem to cause metastasis. it is conceivable that downstream mutations in EGFR signaling might contribute to epidermal to mesenchymal transition (EMT) associated with an invasive or metastatic phenotype. *RAS* activation is important for cellular movements [44, 45]. One should also consider that invasiveness is a mandatory but not sufficient condition for metastasis. The latter requires that in addition to features occurring during invasion, e.g. loss of cell contacts, cytoskeletal deformation along with cellular motility, extracellular matrix degradation, metastasis formation also needs successful attaching and outgrowth of malignant cells in a heterotopic environment. Only a very minor part of malignant cells sheded by the primary tumor site succeed in this complex process. This might give an idea why *KRAS* mutations are frequent in colorectal cancer and may promote local invasiveness but probably do not determine the fate of metastasis. If mutations in the EGFR signaling downstream of *KRAS* are relevant in this process remains to be determined.

## Conclusions

*KRAS* mutation status is significant concordant between areas of the primary tumor and the corresponding metastasis in patients with advanced rectal cancer treated preoperatively with CRT. Thus, our data suggest that sampling either the primary (pre- or posttherapeutical tumor tissue) or metastatic lesion may be valid for the initial evaluation of *KRAS* mutation status for predicting the response to anti-*EGFR* treatment and guiding clinical decisions. These findings should be validated in additional studies with larger sample sets to confirm our data.

## Supporting information

S1 Table Matching base table. Data set for the concordance analysis of *KRAS* mutation status between primary and matched metastatic lesion.
(XLSX)

S2 Table Survival base table. Data set for the *KRAS* mutation status and survival analyses.
(XLSX)
